# Analyzing Instagram Posts on Hypothyroidism: Characteristics, Information Types, Quality, and Reliability

**DOI:** 10.7759/cureus.47132

**Published:** 2023-10-16

**Authors:** Nirmal Patel, Aruna Anantharaj, Roopa Kodimyala, Adik Umeshkumar Patel, Manasi Rane, Anju Maria Thomas, Manisha Bandamede

**Affiliations:** 1 Internal Medicine, St. George’s University School of Medicine, True Blue, GRD; 2 Internal Medicine, Wuhan University School of Medicine, Hubei, Wuhan, CHN; 3 Pathology, Licking Memorial Health Systems, Newark, USA; 4 Internal Medicine, Texila American University, Georgetown, GUY; 5 Internal Medicine, Smt. Nathiba Hargovandas Lakhmichand (NHL) Municipal Medical College, Ahmedabad, IND; 6 Internal Medicine, Thirumala Devaswom (TD) Medical College Hospital, Alappuzha, IND; 7 Family Medicine, Ross University School of Medicine, Bridgetown, BRB

**Keywords:** social media, reliability, quality, discern, global quality scale, gqs, instagram, hypothyroidism

## Abstract

Background and aims

In the age of social media, a vast amount of information is widely and easily accessible. Platforms such as Instagram allow its users to post pictures and videos that can reach millions of users. This could be utilized by healthcare providers to provide education to a vast number of the population about a disease such as hypothyroidism with an easily digestible infographic. However, this easy accessibility comes with the risk of rampant misinformation. This study aimed to evaluate the characteristics of Instagram posts, the type of information, and the quality and reliability of information posted about hypothyroidism.

Methodology

This is a cross-sectional observational study that was conducted over the course of days on Instagram. Top posts meeting inclusion criteria under seven different hypothyroidism-related hashtags were surveyed for content and social media metrics by the authors utilizing Google Forms. The quality and reliability of the posts were analyzed using the global quality scale and DISCERN scales, respectively. The data were exported to an Excel sheet and analyzed using the SPSS software version 21.0 (Armonk, NY: IBM Corp.).

Results

A total of 629 posts met the inclusion criteria of which 62.5% were images and 37.5% were reels. The content heavily focused on the medical aspect of hypothyroidism with posts about symptoms (46.1%), prevention (39.59%), cause/etiology (36.41%), and treatment (34.34%). The median DISCERN score which reflects the reliability of the posts uploaded was highest for doctors at 3 and the least reliable posts were uploaded by dieticians, naturopathic doctors, and patients. This study found that the quality of posts uploaded by nutritionists and naturopathic doctors with a median Global Quality Score (GQS) score of 3.

Conclusions

There is a need to establish a quality control body that regulates the quality and reliability of the posts to curb misinformation and help patients gain easy access to reliable resources that will aid their decision-making.

## Introduction

Low thyroid hormone levels produce hypothyroidism, which has a range of origins and symptoms. When hypothyroidism is untreated, mortality and morbidity rates increase [1-3]. Although a shortage of iodine in the diet is the most common cause of hypothyroidism globally, an autoimmune thyroid condition (Hashimoto thyroiditis) is the most common cause in the United States [4,5]. Levothyroxine must be used every day as a lifelong treatment for hypothyroidism [6,7].

Instagram was founded in 2010 and is an online social network for sharing images and videos. On Instagram, users can submit information to a feed that is viewable by others for 24 hours, use private chat, tag content with searchable hashtags, include multiple photos or videos in a single post, and use the stories feature. Instagram's visual emphasis distinguishes it from different social networking platforms that are perhaps more text-focused, and it might influence how educators utilize Instagram in comparison to other social networking sites [8].

This is a tool used by healthcare professionals to educate patients, disease survivors to post about their ailments, nutritionists to discuss diet, and physical trainers to offer weight reduction advice for conditions like hypothyroidism. Misinformation is always a possibility with such easy access to social media [9]. In this context, this study evaluates the quality and reliability of information about hypothyroidism provided on Instagram.

This study aimed to evaluate the attributes of posts on Instagram, the type of information being conveyed, and the criteria for gauging the quality and reliability concerning content related to hypothyroidism. The quality was assessed using the Global Quality Score (GQS) while reliability was assessed using the DISCERN reliability score.

## Materials and methods

The present study is a cross-sectional observational study conducted over a span of two days, from August 3, 2023, to August 5, 2023, utilizing the social media platform Instagram as its data source. The central objective of the study was to examine and analyze posts related to hypothyroidism within this specific timeframe. To execute this, each participating author was assigned one of seven designated hashtags, encompassing terms such as "#hypothyroid," "#hypothyroidism," "#hypothyroiddiet," "#hypothyroidweightloss," "#hypothyroidfighter," "#hypothyroidsucks," and "#hypothyroidismdiet." Collectively, these authors evaluated a comprehensive total of 1050 posts, with each contributor assessing approximately 150 posts, focusing on those deemed as top posts by the platform's algorithms.

The study adopted a set of inclusion and exclusion criteria to ensure the relevance and quality of the gathered data. Specifically, inclusion criteria encompassed posts presented in the English language, accompanied by titles, which pertained to the theme of hypothyroidism. Conversely, posts were excluded if they deviated from these criteria, such as those employing languages other than English or those unrelated to the context of hypothyroidism.

Analysis of the posts was executed through a comprehensive examination of their diverse characteristics. These attributes encompassed the format of the post, distinguishing between an image and a video, and quantitative metrics such as the number of comments, likes, and follower count of the post's uploader. Moreover, the type of uploader was classified, including categories such as doctors, nutritionists, dietitians, naturopathic doctors, and patients. The content of the posts was further looked into, encompassing a range of topics from symptoms, prevalence, etiology, prevention, diagnosis, treatment, mortality, rehabilitation, and support groups. Notably, the study delved into whether the posts involved patients or their parents sharing personal experiences related to the disease. Additionally, it assessed the presence of promotional content from pharmaceutical companies or doctors.

Intriguingly, the study also undertook an evaluation of the quality and reliability of the posts. This evaluation was facilitated through the utilization of the Global Quality Scores (GQS), score range of 1-5, and DISCERN reliability scores, score range of 1-4, providing a comprehensive overview of the credibility of the information being disseminated.

The DISCERN reliability score is concerned with the dependability of the upload source. The standards, which ranged in scores from 1 to 4, included (A) Were the goals clear and achieved? (a) Is a reliable source of information used? (c) Is the supplied information balanced and unbiased? (d) Are there any other resources listed for the patient's reference? A score of 1 indicated a lack of information, a score between 2 and 3 indicated some information, and a score of 4 indicated all available information [10].

The Global Quality Score (GQS) is measured based on the quality, flow, relevant information, and usefulness to the patient. A score of 1 indicates poor quality, flow, most information missing, and not useful to the patient while a score of 5 suggests excellent quality and flow, contains all the relevant information, and is very useful to the patient [11].

The collected data was subsequently organized and exported to Microsoft Excel for further refinement and organization, ultimately paving the way for subsequent in-depth analysis. The statistical analysis was conducted using the SPSS software version 21.0 (Armonk, NY: IBM Corp.).

## Results

The study analyzed a total of 1050 posts under seven specific hashtags concerning hypothyroidism, with 629 included in the final evaluation, representing a 59.9% inclusion rate. Table 1 describes the hashtags evaluated which included the following: #hypothyroiddiet, #hypothyroidiweightloss, #hypothyroidism, #hypothyroidismsucks, #hypothyroidfighter, #hypothyroid, and #hypothyroidismdiet. Among these, the hashtag "#hypothyroidfighter" had the highest inclusion rate of 66.7%, whereas "#hypothyroidismsucks" had the lowest rate at 44.7%.

**Table 1:** Total posts evaluated and included in the study.

S. no.	Hashtag	Posts analyzed	Posts included
1	#Hypothyroiddiet	150	96
2	#Hypothyroidiweightloss	150	98
3	#Hypothyroidism	150	72
4	#Hypothyroidismsucks	150	67
5	#Hypothyroidfighter	150	100
6	#Hypothyroid	150	101
7	#Hypothyroidismdiet	150	95
Total		1050	629

Table 2 provides insights into the characteristics of the posts, including the type, total audience reached, and the professional background of the uploader. Images constituted 62.5% of posts, with videos or reels comprising the rest 37.5% (n=236). The posts reached a broad audience, with a total of 1,933,357 likes, 13,831 comments, and reaching 26,941,462 followers. The distribution of uploaders presented a varied landscape, including doctors (7.8%), dieticians (21.3%), nutritionists (7.0%), naturopathic practitioners (11.3%), naturopathic doctors (2.7%), patients (14.1%), and others (35.8%).

**Table 2:** Characteristics of posts.

Type of posts		Values
Image		393 (62.5%)
Video/reel		236 (37.5%)
Total number of audiences reached	Number of likes	1,933,357
	Number of comments	13,831
	Number of followers	26,941,462
Type of uploader	Doctor	49 (07.8%)
	Dietician	134 (21.3%)
	Nutritionist	44 (07.0%)
	Naturopathic practitioner	71 (11.3%)
	Naturopathic doctor	17 (02.7%)
	Patient	89 (14.1%)
	Others	225 (35.8%)

Table 3 enumerates the type of information shared and was systematically classified into 14 specific categories, reflecting the diverse facets of hypothyroidism. Key findings from the analysis reveal that descriptions of symptoms were most prevalent, occurring in 46.1% (n=290) of the posts. Information about the cause/etiology and prevention were also notable, found in 36.41% (n=229) and 39.59% (n=249) of posts, respectively. Additionally, the study identified that 60.25% (n=379) of the posts consisted of digitally created content, and 10.81% (n=68) contained promotional material by pharmaceutical companies or medical professionals.

**Table 3:** Type of information shared.

Criteria	N (%)
Description of the disease? (Explaining what is it)	209 (33.23%)
Description of symptoms	290 (46.1%)
Information about prevalence/incidence?	32 (5.09%)
Information about cause/etiology?	229 (36.41%)
Info about diagnosis	159 (25.28%)
Info about prevention	249 (39.59%)
Info about treatment	216 (34.34%)
Information about mortality	5 (0.79%)
Information about rehabilitation	69 (10.97%)
Information about support groups	43 (6.84%)
Info about people/patients sharing their own experience	132 (20.99%)
Info about parent sharing their experience with their family members	4 (0.64%)
Is it a digitally created image/video?	379 (60.25%)
Does the post have promotional content by pharmaceutical companies or by doctors?	68 (10.81%)

Table 4 meticulously assesses the quality and reliability of posts related to hypothyroidism by utilizing the Global Quality Score (GQS) and the DISCERN method, respectively, across different uploaders categories, including doctors, dieticians, nutritionists, naturopathic practitioners, naturopathic doctors, patients, and others. The Kruskal-Wallis test yielded significant p-values (<0.001) for both the Global Quality Score and reliability score, suggesting significant variations in the quality and reliability across different types of uploaders. For instance, doctors emerged with the highest median quality and reliability scores (GQS median of 3; Q1, Q3: 2.5, 3.5; DISCERN median of 3; Q1, Q3: 2, 3), while dieticians, naturopathic doctors, and patients demonstrated the lowest median reliability score of 1.

**Table 4:** Assessment of quality and reliability of posts based on uploader. Values are written as median (Q1, Q3) where Q is Quartile. P-value < 0.05 is significant.

Variables	Global quality score median (Q1, Q3)	Reliability score (DISCERN) median (Q1, Q3)
Doctor	3 (2.5, 3.5)	3 (2, 3)
Dietician	2 (1, 3)	1 (1, 2)
Nutritionist	3 (2, 3.75)	2 (1, 2)
Naturopathic practitioner	3 (2, 3)	2 (1, 2)
Naturopathic doctor	2 (1, 3)	1 (1, 2)
Patient	2 (1, 3)	1 (1, 2)
Others	2 (1, 3)	2 (1, 2)
P-value (method: Kruskal-Wallis test)	<0.001	<0.001

## Discussion

To study the quality and reliability of posts related to hypothyroidism on Instagram, a day study was conducted wherein all authors analyzed 100 posts each. After meeting the inclusion and exclusion criteria, a total of 629 posts were taken into consideration for this study.

The important findings in this study were the following. The total number of likes was 1,933,357, comments were 13,831, and followers were 26,941,462. In another study on coronavirus disease 2019 (COVID-19) misinformation on Instagram, 19 or 37% of uploaders were physicians with a total number of followers being 2,567,971 and an interquartile range (median) of 11,000 [12].

In this study, 7.8% of posts were uploaded by doctors, 21.5% by dieticians, 7.0% by nutritionists, 11.3% by naturopathic practitioners, 2.7% by naturopathic doctors, 14.1% by patients and 35.8% by others. Whereas, in another study on dermatology content on Instagram by Quijote et al., 226 or 48.1% of post creators were identified as healthcare providers [13]. Board-certified dermatologists made up the majority of the healthcare providers uploading these posts on Instagram, making up 154 or 64.4% of them [13]. Other popular Instagram posters included businesses/industries, laypersons, influencers, and advocacy or charitable organizations [13].

In this study, 209 posts had information describing hypothyroidism, 290 posts described its symptoms, 229 posts were on its cause/etiology, and 216 posts were on its treatment. Whereas in another study on seizure knowledge circulation on Instagram by Popoola-Samuel et al., 163 posts had information about the symptomology of seizures, 66 posts described the etiology of the disease, and 86 posts provided treatment options [14].

In this study, the mean Global Quality Score (GQS) of posts uploaded by doctors was 3, by dieticians was 2, by nutritionists was 3, by naturopathic practitioners was 3, by naturopathic doctors was 2, by patients was 2, and by others was 2. Whereas in another study on seizure knowledge circulation on Instagram by Popoola-Samuel et al., the majority (33.9%) of the GQS of the posts uploaded was a 3. A score of 1 was given for 30.4% of the posts, a score of 2 was given for 24.6% of the posts, a score of 4 was given for 10.4% of the posts, and a score of 5 was given for 0.7% of the posts.

In this study, the mean reliability score (DISCERN) of posts uploaded by doctors was 3, by dieticians was 1, by nutritionists was 2, by naturopathic practitioners was 2, by naturopathic doctors was 1, by patients was 1, and by others was 2. Whereas in another study on oral cancer information on Instagram by Passos et al., the concern of posts uploaded by content creators was mostly of low reliability (66.7%). High-reliability content made up 25.6% of the posts, and 2.6% of posts were of moderate reliability [15].

Limitations

There were a few limitations in our study that must be addressed. Firstly, there were a total of only seven hashtags that were used for analyzing the posts, which places a restriction on the breadth of content covered in the posts. Moreover, only the top 100 posts relevant to each hashtag were analyzed for this study. As such, other older posts were excluded because a user cannot view all of the posts available on Instagram.

## Conclusions

In conclusion, social media platforms such as Instagram contain numerous daily uploads, which make it difficult to ascertain the quality and reliability of the information that is shared. Content creators come from different backgrounds and have their own individual motivations or goals when they upload posts for users to view. Consequently, in the future, there should be strict regulation on the type of information, its quality, and its reliability by medical bodies and government authorities to filter out the circulation of information with low quality and reliability. In the long run, this implementation by Instagram and other social media platforms will help improve patient education and their own decision-making. At the end of the day, however, Instagram posts are not a replacement for years of medical experience, and users should continue to seek professional medical assistance for their ailments or queries.
